# Supplementary material for: Are carbon emissions trading and green financial instruments synergistic? -Comprehensive quantitative research based on content analysis
Source: PLoS One. 2024 Mar 7;19(3):e0298601. doi: 10.1371/journal.pone.0298601 (PMC10919601; doi:10.1371/journal.pone.0298601)
Supplement: S1 Data — (ZIP) [file pone.0298601.s001.zip › Data/地级市Z_F工作报告.zip!/╜¡╦╒/─╧═¿╩╨/└┤╫╘╣1⁄2╓┌║┼í╛┬φ┐╦╩2╛▌═°í┐_2007.docx]

各位代表：

　　现在，我代表市人民政府向大会作工作报告，请予审议，并请市政协各位委员和其他列席人员提出意见。

　　一、"十一五"开局良好

　　2006年，在省委、省政府和市委的正确领导下，全市上下坚持以科学发展观统领经济社会发展全局，锐意进取，奋力拼搏，较好完成了市十二届人大四次会议确定的各项目标任务，巩固提升了跨越发展、科学发展、和谐发展态势，实现了"十一五"发展的良好开局。

　　（一）经济增长速度继续保持全省领先，发展质态持续提升。全市预计实现地区生产总值1758.34亿元，按可比价计算增长15.7%，增幅连续两年全省领先。财政总收入217.57亿元、增长27.1%，占地区生产总值12.4%、比上年提高0.8个百分点；地方一般预算收入92.53亿元，增长28.5%，增幅高于全省平均水平3.4个百分点。产业结构发生较大变化，三次产业结构为9.6∶56∶34.4，二、三产业占比首次超过90%。规模以上工业增加值增长22.7%，增幅居全省第二；工业经济效益指数增幅、工业产销率均居全省首位。服务业增加值增长15.7%，2003年以来首次达到地区生产总值增幅。科技创新、资源节约、环境保护进一步加强。全社会研发投入占地区生产总值比重首次超过1%，高新技术产业产值占规模以上工业总产值比重超过20%。耕地总量连续15年实现占补平衡。主要污染物化学需氧量和二氧化硫排放量分别削减3%和6%。区域发展更趋协调，南三县（市）持续在高平台上较快增长，北三县（市）总体保持发展速度领先优势。南通在长三角16个城市中综合竞争力排名由上年第14位前移至第7位。

　　（二）江海联动开发取得重大进展，大交通新格局加速形成。江海联动开发26项重点工作基本完成。新增沿江港口岸线3.5公里。狼山港三期2个10万吨级通用散货泊位投入试生产。南通港货物吞吐量1.04亿吨，跻身全国沿海亿吨大港行列。沿江装备制造业等临港新兴产业加速形成。中远川崎二期、熔盛造船、振华港机等39个重点项目开工建设，醋纤四期、扬子高丽钢线、绿山集成电路等42个重点项目竣工或部分竣工投运，总投资158.3亿元的王子制纸项目获国务院批准。洋口港开发进入全面实施阶段，陆岛通道接岸引堤工程通过验收，黄海大桥全面开工，江苏液化天然气项目上报国家发改委审核。如东风电一、二期10台机组并网发电，填补江苏空白。江海旅游业加速发展。江海联动开发推动了有效投入的持续增长和投资结构的优化。全市完成全社会固定资产投资1048.9亿元。其中，规模以上高新技术产业投入增长35.1%，规模以上服务业投入增长33.5%，分别高于全社会规模以上投入增幅5.7个和4.1个百分点。重大交通基础设施建设加快推进。苏通大桥全面进入主桥主跨合龙施工阶段，崇启大桥项目建议书通过国家发改委批准，崇海大桥立项工作启动，南通火车站主站房建设基本完成，南通至南通东铁路完成铺轨前期工程，沪通铁路项目前期工作进展顺利。

　　（三）外资民资再攀新高，发展活力和动力不断增强。成功举办港洽会等系列重大招商活动，开放型经济实现历史新突破。利用外资连续4年"撑杆跳"，全年新增工商登记注册外资69.39亿美元，增长123.2%；注册外资实际到账27.44亿美元，增长79.1%，跻身全国大中城市利用外资前十强；利用外资质量明显提升，新批超千万美元项目293个，其中近亿及超亿美元项目8个，新批项目平均单体规模647万美元，比上年提高27.4%。进出口总额100.62亿美元，增长18.1%。完成对外劳务承包营业额7.39亿美元，连续11年居全省第一。各级各类开发区主要指标在全省开发区中的排位大幅前移。民营经济继续保持强劲增长态势。个体工商户净增7100家，累计25.17万家、为全省第一。私营企业净增2.34万家，累计9.39万家；注册资本净增485亿元、比上年多117亿元，累计1432亿元、列全省第二。民营经济增加值占地区生产总值45.1%，民资投入占全社会投入75.3%，分别比上年提高2个和3.4个百分点。争创"名企、名品、名人"工作成效突出。新增中国名牌产品9个，居全省第二；新增中国驰名商标1个。建筑业完成施工产值1098亿元，增长29.1%；新增特级资质企业6家，累计12家、占全省一半。体制改革深入推进。国有资产监管和经营业绩考核体系进一步完善，国有资产实现保值增值。市属社会公益型事业单位改革稳步推进。投融资体制改革加快。各金融单位年末本外币贷款余额1233.25亿元，比年初增加208.79亿元。境内外上市企业新增2家，4家上市公司完成股权分置改革。行业协会、商会有序发展。接轨上海工作深入开展。

　　（四）围绕"民富、村美、风气好"目标，新农村建设扎实推进。农业结构调整成效明显。高效农业规模化"133行动计划"开始启动。亩净收益2000元以上农田占比为24.5%，比上年提高5个百分点，占比在全省处于领先水平。农业综合开发和农机装备水平不断提高。农业标准化工作、农产品质量建设取得新成效。

　　项目农业完成投资51.5亿元，增长31.7%；农业利用外资实际到帐2.1亿美元，列全省第一。各类农民经纪人发展到14万人，农村劳动力转移新增8.04万人。农村环境建设进一步加强，镇村布局规划编制完成并率先通过省验收；"百村示范、千村整治"工程和小康示范村建设有效实施；道路通达工程、农民健康工程等"农村新五件实事"完成阶段性任务。

　　（五）城市功能不断提升，城市化进程加快。城市总体规划纲要编制完成。城市建设力度加大，市区通富大桥、滨江大桥等68项工程全面开工，其中通京大道等40多项工程顺利完工，城闸大桥主体部分基本完成，全年完成城建投入45亿元，为历年最多。新区建设快速推进，体育会展中心按期建成并作为省十六运主会场投入使用，园艺博览园开工建设。房地产业健康发展。市区新增绿地280公顷，绿化覆盖率41.8%；濠河综合整治与历史风貌保护工程获得中国人居环境范例奖，我市获"全国水环境治理优秀范例城市"称号。市区新增日供水能力20万吨、日污水处理能力7.5万吨。继续推进"五城同创"，建立综合整治城市违法建设长效管理机制；市区百日环境整治771个项目全部按时完成。我市建成"国家环境保护模范城市"，在"五城同创"中率先突破，并被授予"保护臭氧层示范市"称号。统筹推进中心城市与城镇建设，县（市）城、中心镇基础设施建设快速推进，城市化率48%，比上年提高2.2个百分点。

　　（六）社会事业协调发展，精神文明和民主法制建设进一步加强。科教事业取得新成绩。12项科技成果获省科技进步奖，列全省第一；专利授权量实现倍增，成为国家知识产权试点城市。教育现代化工程大力推进，76%的乡镇通过验收；合格学校建设加快，在全省率先完成农村初中四项配套工程建设任务；基础教育水平保持全省领先，高考总、均分等12项指标列全省第一；60%的乡镇成人教育中心学校建成特色学校；职业教育、高等教育进一步发展，3所高职院校通过全国高职高专人才培养工作水平评估。"人才特区"试点工作全面启动，人才环境不断优化。公共卫生服务体系建设进程加快，农村以行政村为单位基本实现社区卫生服务全覆盖，乡镇卫生所规范化建设提前完成。群众性文化活动积极开展，"百千万"工程深入推进。文化产业发展、历史文化保护取得新成绩。广播电视事业进一步拓展，新增农村有线电视用户18万户，居全省首位。农村体育健身工程全面启动，60%的行政村达到省标准；省运会获得金牌数首次跻身前三甲。人口与计划生育工作继续加强，人口连续9年负增长。全市第二轮地方志编纂工作全面启动。精神文明建设取得新成效。如皋"爱心邮路"被作为实践社会主义荣辱观重大典型、全国首家政府命名"无红包医院"市第三人民医院先进经验在全国推广。民主法制建设进程加快。政务公开范围进一步扩大。"市长热线"、"市长信箱"工作机制进一步完善。基层民主建设得到加强。"四五"普法工作受到省委、省政府表彰。切实维护群众合法权益，信访总量、集访批次双下降。深入推进新一轮"平安南通"建设。大力加强社会治安综合治理，社会矛盾纠纷大调解机制和社会治安大防控体系进一步完善，各类纠纷调解成功率97.6%，人民群众对社会治安的满意率98.6%、居全省第一，被省委、省政府授予"社会治安安全市"称号。食品、药品安全监管工作得到加强，整顿规范市场经济秩序工作和社会信用体系建设不断强化。国防教育、征兵、民兵预备役、"双拥"、优抚安置、人防、国家安全工作取得新成绩。外事、侨务、台湾事务、民族、宗教、审计、统计、机关事务管理、气象、防震、无线电管理、档案、保密、新闻出版、哲学社会科学等工作进一步加强。

　　以一流精神面貌、一流城市形象、一流组织服务、一流参赛成绩，成功承办了省运会历史上规模最大、设置项目最全、参赛人数最多的一次体育盛会。筹办省十六运中激发出的"举市一致、团结拼搏，高点定位、争创一流，大胆探索、勇于创新，勤奋敬业、无私奉献，文明向上、和谐奋进"的精神，将激励我们加速推进全面小康、全面腾飞宏伟事业。

　　（七）富民进程继续加快，社会保障得到强化。城镇居民人均可支配收入14058元、增长13.5%，预计农民人均纯收入6100元、增长11%，城乡居民收入连续3年保持两位数增长。年末城乡居民储蓄存款余额1373.4亿元，增长15.3%。市区居民消费价格总指数为101.7%。城镇新增就业6.9万人，年末城镇登记失业率为3.2%、低于全省水平。社会保障覆盖面继续扩大，市区基本养老保险基金实现当期收支平衡。企业退休人员基本养老金标准、企业最低工资标准、优抚对象抚恤补助标准均得到提高。城乡低保继续全覆盖。农民工工资和被拆迁、被征地农民补偿款全部发放到位。社会救助不断强化，各级财政普遍增加了助困、助学、助老、助残投入，农村大病医疗救助全面启动；农村五保户集中供养率55.7%，比上年提高4个百分点。各类安全生产事故起数和死亡人数连续5年实现双下降。老龄事业持续发展，职工权益维护、妇女、儿童、青少年、残疾人、关心下一代、老区开发、见义勇为、红十字和慈善等工作进一步加强。

　　（八）认真办好实事项目，既定任务全面完成。⑴全年提供就业岗位22万个，其中市区11.8万个，超出计划的83.3%和195%；免费开展农村劳动力转移培训7.4万人、农业实用技术培训30.8万人、创业培训1.7万人，均超出年度计划。⑵全市新型农村合作医疗覆盖面91.3%，高出计划6.3个百分点。⑶全市城乡义务教育阶段学生秋季入学全部免收学杂费，为7.1万名学生免费提供教科书，为1.1万名寄宿生补助生活费191万元。⑷启海区域供水一期工程竣工，西北片区域供水工程完成投资1.2亿元。⑸疏浚市级河道3条段、县级河道24条段、乡级河道208条段，共清淤土方2700万方，超出计划的22.7%；集中整治市区河道12条段。⑹改建、扩建农村桥梁500座；新建和改造农村公路750公里，超出计划的114.3%。⑺市区老年活动中心建成，市老年大学办学条件有所改善，市区新增社区老年活动场所10个。⑻市区残疾人综合服务中心建成；向3000名盲人捐赠盲杖，向2000名下肢残疾人捐赠轮椅。⑼市区优化公交线路10条，新增和改造公交站台（点）67个，超出计划的34%。⑽对市区17个老住宅片区实施以雨污分流、水系贯通为重点的综合改造，为计划的5.7倍；市区新建低价位商品房88万平方米，超出计划的46.7%；安排3020万元为市区住房困难家庭提供政策性住房保障。⑾市区农贸市场新建和改造项目完成计划任务。⑿市区人民公园、文峰公园、唐闸公园、滨江公园免费向市民开放。

　　各位代表，过去的一年，我们坚持把推进跨越发展作为全面落实科学发展观的第一要务，强化确保"十一五"首战必胜的目标责任和关键措施，持续猛攻有效投入，重大基础设施、重点产业、重要民生项目建设等106项重点工作完成情况良好。坚持把转变经济增长方式作为全面落实科学发展观的中心环节，坚决贯彻中央宏观调控政策措施，以有效投入促进结构优化、产业升级，以布局集中推动产业集聚、发展集约，以降耗减排促进资源节约、环境保护。坚持把改革开放作为全面落实科学发展观的体制保障，着力突破体制机制瓶颈，破解发展难题。坚持把构建和谐社会作为全面落实科学发展观的重要任务，健全突发公共事件预警和应急处理机制，努力解决好人民群众最关心、最直接、最现实的利益问题。坚持把加强自身建设作为全面落实科学发展观的内在要求，创新服务和管理方式，优化行政审批，完善机关"三位一体"的考核机制，提高行政管理效能，营造真抓实干的良好氛围；认真做好基础性、长远性工作，组织开展50多项系列调研，找准工作着力点，提高抓落实的成效；积极推进依法行政，规范行政执法行为；加强政风行风建设，深入推进反腐倡廉工作。过去的一年，我们更加自觉地接受市人大及其常委会的法律监督、工作监督和市政协的民主监督，认真执行人大及其常委会决议并及时报告工作，支持政协履行职能，切实抓好人大议案、政协建议案和人大代表建议、政协委员提案的办理工作，市人大代表和政协委员满意基本满意率分别为96.7%、96.9%。

　　各位代表，过去一年所取得的成绩，是在省委、省政府和市委的正确领导下，在市人大、市政协的监督支持下，在历届政府工作的基础上，全市上下务实奋进、开拓创新的结果，凝聚着广大创业者、劳动者、建设者的辛劳和智慧。在此，我谨代表市政府向广大干部群众，向人大代表、政协委员和离退休老同志，向人民解放军、武警驻通部队官兵和公安干警，向各民主党派、工商联、各人民团体和各界人士，向支持南通现代化建设的部、省驻通单位，向所有关心南通发展的港澳台同胞、海外侨胞和国际友人，表示崇高的敬意和衷心的感谢！

　　各位代表，我们也清醒地看到，全市经济社会发展中还存在许多矛盾和问题，主要是：人均地区生产总值还低于全省平均水平；产业层次还不高，结构性矛盾仍然比较突出；经济增长方式的转变还不够快，节能降耗、污染减排的任务还很艰巨；农村基础设施建设滞后，农村生态环境有待进一步改善，农民持续增收难度加大；城乡就业结构性压力尚未得到根本缓解；社会弱势群体生活还比较困难，一些涉及群众切身利益的问题有待进一步解决。政府自身建设中还存在一些薄弱环节，政府职能转变任务还很重，社会管理和公共服务需要进一步加强。对此，我们将采取切实措施，认真加以解决。

　　二、2007年工作目标与总体思路

　　今年是党的十七大召开之年，是全面落实省、市党代会精神的开局之年，也是加快推进全面小康、全面腾飞的关键一年。当前国内外宏观发展环境、南通自身多年积蓄的发展基础和业已形成的强劲发展态势、江海联动开发和大交通格局即将根本改变等诸多比较优势，对做好今年各项工作、加速"全面达小康、建设新南通"进程十分有利。我们一定要倍加珍惜千载难逢的历史机遇，倍加珍惜来之不易的发展环境，倍加珍惜政通人和的良好局面，上下同心，乘势而上，按照省委、省政府希望南通成为全省全面达小康的新动力、建设新江苏的新优势、"两个率先"的新空间的战略要求，把跨越发展、科学发展、和谐发展的良好态势巩固好、延续好，把人民群众的切身利益维护好、发展好。与此同时，我们不可忽视国内外经济环境不确定因素的影响，不可忽视当前经济社会生活中一些突出矛盾对经济增长、社会发展的制约，居安思危，未雨绸缪，全力开创又好又快发展新局面。

　　今年政府工作的指导思想是：以邓小平理论和"三个代表"重要思想为指导，全面贯彻科学发展观和构建社会主义和谐社会的战略思想，认真落实党的十六大、十六届三中、四中、五中、六中全会，省第十一次、市第十次党代会和中央、省、市经济工作会议精神，紧紧围绕实现全面小康、推进全面腾飞的主题，更加注重扩张规模与提升质量的有机统一，更加注重适应宏观调控与化解发展瓶颈制约的有机统一，更加注重加快发展与关注民生、保护生态的有机统一，更加注重创业、创新、创优与群众增收、企业增利、财政增长的有机统一，更加注重三个文明建设与构建和谐社会的有机统一，保持全市经济发展速度效益全省双领先，努力实现高平台上持续又好又快发展，促进社会和谐，以优异成绩迎接党的十七大召开。

　　今年经济社会发展的主要预期目标是：地区生产总值增长15%左右、力争突破2000亿元，地方一般预算收入增长20%左右，单位生产总值能耗下降4%左右，主要污染物化学需氧量和二氧化硫排放量分别削减3%和6%，全社会固定资产投资增长20%以上，注册外资实际到帐增长20%左右，社会消费品零售总额增长15%以上，城镇居民人均可支配收入增长11%以上，农民人均纯收入增长10%以上，城镇登记失业率控制在4%以内，人口自然增长率控制在零以下。

　　按照上述思路和目标，今年政府工作将注意把握好以下几个方面：

　　--紧紧抓住大交通格局根本改变带来的历史机遇，不断提高城市综合竞争力。按照构建沪苏通金三角、建设"北上海"的战略定位和"经济增长走在江苏前列"的目标，准确把握经济国际化加速和南通工业化提升的阶段性特征，抢抓苏通大桥南北贯通、沪通铁路和崇启大桥开工、崇海大桥项目加速推进、区域枢纽地位加速形成带来的历史机遇，坚定不移加快新型工业化、城市化、市场化、经济国际化进程，充分放大改革率先、开放带动、江海联动优势，有效承接上海和苏南辐射，加速推进全面小康、全面腾飞，提高在全省发展中的份量和贡献，提高在长三角区域中的经济战略地位。

　　--突出资源节约、环境保护和自主创新，加快优化经济结构和转变增长方式。按照"科学发展走在江苏前列"的目标定位，坚持科教优先、环保优先、节约优先，以加快发展集约型、集群型、开放型、内生型、港口型、都市型经济为取向，好中求快、优中求进，通过科技创新增强核心竞争力，通过产业升级缓解资源环境压力，通过集聚、集中、集约实现清洁增长、节约增长、高效增长，确保完成节能降耗和污染减排的约束性目标，加快建设创新型城市和资源节约型、环境友好型社会。

　　--坚持以人为本、富民优先，把和谐社会建设贯穿于改革发展全过程。按照"和谐社会建设走在江苏前列"的目标定位，把社会发展和改善民生放在更加突出的位置，加强社会事业建设，强化利益关系调节，增强社会创造活力，促进社会公平正义。大力推进公共产品和基本公共服务均等化，加快构建公共财政体制框架。调整财政支出结构，新增财力主要用于新农村建设、社会事业发展和社会保障支出。同时，把公共资源更多向经济薄弱地区、社会弱势群体和发展薄弱环节等方面配置，着力解决人民群众最关心、最直接、最现实的利益问题，努力让人民群众更多享受改革发展成果。

　　三、全力推进经济又好又快发展

　　（一）着力推进产业结构优化升级，加快新型工业化进程。从提升自主创新水平、放大规模集聚效应、强化能源资源节约入手，推动制造业和服务业良性互动发展，提高产业层次。以发展高新技术产业为重点，大力发展先进制造业。持续推进工业争先进位，力争全部工业增加值达到1000亿元。围绕构建"一特多强"的产业格局，重点放大船舶修造及配套产业特色，加快建设船舶配套产业集中区、海洋工程配套产业园；着力推进依托海港的电力、石化等重化工业发展，培育新的产业竞争优势；运用先进适用技术改造提升传统制造业，做强做优现代纺织、轻工食品等优势产业。坚持信息化带动工业化，大力发展电子信息、光机电、新材料、生物医药、软件等高新技术产业，力争高新技术产业产值增长28%。着眼产业集聚，重点跟踪和推进13个省级以上开发区和50个工业集中区建设，重点促进20个特色经济板块发展壮大，加快推进市区企业退城进园搬迁改造。实施重点企业梯度培育工程，力争销售收入超亿元工业企业达到550家，其中超10亿元企业35家。综合运用规划、政策、服务、财政资金等手段，促进资源要素加速向支柱产业、优势行业、重点企业倾斜和集中。强化工业经济运行监控，最大限度减少相关因素对工业生产的不利影响。加速打造全国一流建筑强市，培育产值超100亿元企业集团2个、超50亿元企业集团5个。以全面提速和集聚区建设为重点，提高现代服务业贡献份额。紧紧抓住服务业全面对外开放的机遇，加快构筑现代服务业发展新格局，力争服务业增加值增长15.5%以上。在巩固提高传统服务业的同时，加快发展现代物流、商务服务等生产性服务业。加大服务业招商引资力度，重点培育和引进一批现代物流企业，大力发展第三方、第四方物流。积极培育金融保险、信用担保、信息咨询等各类商务服务业，全力突破会展业。进一步拓展旅游市场，大力发展旅游业。积极有序发展房地产业，保持房地产市场的持续健康发展。依托中心城市、重点园区、交通枢纽，以商务区、商业区、现代物流区、休闲旅游区为重点，加快建设一批现代服务业集聚区。强化政策引导，增强消费对经济增长的拉动力。以完善技术创新体系为重点，大力推进自主创新。围绕争创国家级高新技术创业服务中心，建立健全以企业为主体、市场为导向的技术创新体系，抓好特色产业基地、科技孵化器、公共服务平台、工程技术研究中心（重点实验室）、企业技术中心等创新载体建设。深入推进产学研合作，实施产学研科技成果转化项目200项。大力培育重大科技成果转化项目，加速科技成果产业化。以国家知识产权试点市建设为契机，提高引进消化吸收再创新能力。加大科技创新创业政策的落实力度，进一步优化自主创新环境。全力抓好省人才特区试点，加快培养引进创新人才，努力形成一支高层次、领军型科技人才队伍。以发展循环经济为重点，提高资源要素利用效率。完善并实施循环经济发展规划，全面推行清洁生产，强化节能、节地、节水、节材和资源综合、循环、高效利用，加快形成节约型产业结构和生产方式。完善能耗统计和发布制度，抓好六大重点领域、五大重点行业和百家重点企业节能降耗工作，建立新上项目能耗审核制度。大力实施节能重点工程和资源综合利用示范项目，着力推进墙体材料革新和建筑节能，坚决淘汰高能耗产能。调整能源生产结构，大力发展天然气、风能、太阳能、生物质能等清洁、再生能源。严格耕地保护目标责任，加强土地、岸线等不可再生资源的使用规划和管理。推行工业用地招标挂牌出让，积极探索产业用地二次开发机制，加大闲置土地处置力度。完善资源能源节约激励制度，提高经济增长质量和效益指标考核比重。

　　（二）着力加大城乡统筹力度，加快新农村建设步伐。全面落实工业反哺农业、城市支持农村和多予少取放活的方针，以促进农民增收和加强农村投入为重点，以实施省"新农村建设十大工程"和"农村新五件实事"为主要抓手，深入推进"民富、村美、风气好"的新农村建设。积极争创全省高效农业规模化第一市。把建设现代农业作为推进新农村建设的首要任务，坚持不懈推进农业结构调整，大力实施高效农业规模化"133行动计划"，确保亩净收益2000元以上农田占比提高到30%。继续大力发展项目农业、循环农业，力争"三资"开发农业投入超过50亿元。积极推进农业标准化，提高无公害、绿色农产品比重。运用现代科技武装农业，注重农业科技队伍建设，提高农业装备水平，强化农业综合开发，健全农技推广体系。加快农业产业化进程，增强农业龙头企业带动力。深化农村综合改革。扩大乡镇机构改革试点。落实各项支农惠农政策，提高财政投入用于农村的比重。大力发展农民专业合作组织，推进农村社区股份合作，开展土地股份合作和农业保险试点工作。继续加强农村经营管理，规范"一事一议"筹资筹劳。控制和化解镇村债务，严禁新增不良债务。强化农村基层组织，加快农村公共服务中心建设。大力发展村级集体经济，增强村级综合服务功能。加大对经济薄弱村的帮扶力度。进一步加强农村基础设施建设。加快优化镇村布局，统筹推进农村道路、供水和教育、医疗、文化、体育等基础设施建设。尊重农民意愿，按规划有序推进农民集居区和农村新社区建设。深入实施"百村示范、千村整治"工程，强化以"三清"工程为重点的农村环境综合整治，加快建立以污水和垃圾处理为重点的农村环卫体系。努力让农民得到更多实惠。加强职业培训和继续教育，提高农民就业创业技能和整体素质。完成农业实用技术培训18.6万人次、农村劳动力转移培训5.5万人次和农民创业培训1万人次。继续实施农民收入倍增工程，进一步推动农村劳动力向农外转移，不断增加农民非农收入。

　　（三）着力加速经济国际化和市场化进程，加快培育竞争新优势。进一步加大改革开放力度，保持经济增长活力和效率。加快开放型经济转型升级。以精心筹办第九届亚洲艺术节、第五届省园艺博览会、港口经济洽谈会、第三届世界大城市带论坛、国际友好城市交流大会五大活动为平台，全方位、多层次、宽领域扩大开放。依托大桥大港效应，强化对外推介力度，重点抓好产业集群和产业链延伸招商。加大招商选资力度，重点引进投资强度高、技术含量高、产业带动强、贡献能力强和资源消耗低、污染排放低的项目。进一步完善招商组织、考核体系。加强外商生活服务设施建设，营造更好的投资环境。加快转变外贸增长方式，扶持出口大户和高新技术、自主知识产权、自主品牌产品出口；积极应对贸易磨擦，提高"大通关"效率，力争外贸出口总额增长20%以上。着力提升外经合作层次，全力支持有条件企业开展境外投资和跨国经营，保持外经全省领先地位。积极开展对外交流合作，进一步构建各方参与、充满活力的大外事新机制。加强载体建设，完善各级开发区功能规划，推动共建共用，增强产业集聚和辐射带动能力。深入推进接轨上海工作。在继续做好规划、产业、人才、港口等"硬接轨"的同时，加快推进体制、机制、理念、思路等"软接轨"，不断拓展接轨上海的领域和空间，加速融入长三角经济一体化进程。推动民营经济扩量提质。以私营企业注册资本超2000亿元为目标，继续推动全民创业，力争全市家庭创业率提高到13%以上；以全国500强民企为重点，大力招引市外民资，积极推进民资外资嫁接，力争新增私营企业中公司制企业占比、新增私营企业注册资本中公司制企业注册资本占比和市外民资占比均达到60%。以争创"三名"为抓手，实施规模扩张、自主创新、品牌经营和企业境内外上市四大工程，力争新增营业收入超10亿元私营企业5家、省级以上高新技术企业30家、中国名牌产品和驰名商标10件、上市公司2－3家。继续深化体制改革。健全国有资产营运、管理、监督体制，实现市属国有资产监管全覆盖，确保国有资产保值增值和收益。深化投融资体制改革，完善公共产品投入社会化、市场化机制。加快实施社会公益型事业单位分类改革，大力推进"管办分离"，积极探索政府购买公共服务新机制。继续强化各类要素市场建设，加快发展资本、土地、产权、技术、人才、劳动力等市场。

　　（四）着力推动重大项目突破，加快提升江海联动开发水平。以沿江资源整合和沿海港口开发为重点，进一步释放江海资源优势。坚定不移猛攻有效投入，优化投资结构。主动顺应国家宏观调控要求，更加注重以增量带动调整，确保全社会固定资产投资1200亿元以上，其中规模以上投入突破1000亿元。保持工业投入占比不低于70%，提高高新技术产业和现代服务业投入比重，提高技术改造投入比重，提高规模以上投入特别是亿元以上项目投入比重。重点抓好王子制纸、振华港机、中远川崎二期、熔盛造船等120个市级重点项目建设和50个市级重大产业储备项目前期工作。建立重大产业项目用地直供制度，实施市区招引重大制造业项目和退城进园项目利益补偿机制，加强对落地项目的跟踪评估和增量资本产出率的考核。全力突破重大项目，加速江海开发进程。加快修编完善沿江产业空间布局规划和海洋经济发展规划，编制海域使用规划和海洋环境保护规划，着力抓好洋口港区控制性详规修编和报批工作。坚持开发与保护并重，深入推进沿江岸线整合、整治和开发，重点加快市区岸线和如皋港区石化码头岸线整合，力争启动新通海沙整治开发，加快推进横港沙、长江北支岸线整治开发和如皋中汊、天生港水道整治前期工作。加快洋口港黄海大桥和太阳岛工程建设，基本形成临港工业区项目进区条件，积极推进港区航道整治一期工程，力争洋口港开发建设完成各类投资30亿元。加快吕四港规模开发前期工作。力争全市新增万吨级以上泊位8座，南通港货物吞吐量达到1.2亿吨。坚持以大项目带动大开发，争取江苏液化天然气等一批关键项目获批，加快引进一批龙头型、链条型重特大项目，推动集约开发和产业集聚。乘势而上完善基础设施。实现苏通大桥南北贯通，尽快开工建设崇启、崇海大桥。确保沿江高等级公路全线建成通车，开工建设江（都）海（安）高速，加快建设204国道、334省道快速化一级公路。力争开工建设沪通铁路，抓好铁路"一线三中心"工程续建，争取开通至北京等地始发列车，积极推进洋口港区等铁路专用线前期工作。加快南通机场战略性重组。

　　（五）着力推进中心城市规模扩张和功能完善，加快城市化和城市现代化进程。深度推进"五城同创"，塑造城市特色和个性魅力。进一步强化规划龙头作用。启动第四轮城市总体规划修编，完成城乡规划全覆盖任务。编制完成市域综合交通规划、城市近郊区控制性规划和市区通吕运河、海港引河两岸生态景观概念规划等16项规划。进一步做大做优城市。拉大中心城市框架，完善市区道路网络，力争实现四环环通并打通环内主次干道，启动五环建设。城闸大桥、通富大桥建成通车，滨江大桥完成主体工程。继续做好濠河资源整合和周边绿化景观完善；高标准建成园艺博览园，大力推进五山景区整体开发；加快建设4个市级公园、3条绿化景观带；启动市区通吕运河、海港引河两岸景观带示范段建设。加快新区中央商务区、社会服务区建设，兴建文化活动中心；加快推进苏通大桥桥头区、火车站站前区等重点区域建设。完善提升老城区功能，启动寺街保护性开发，推进老住宅片区和城中村改造。继续强化市区污水收集、处理和供水、停车等公用设施建设。采取综合措施解决交通拥堵重点区域通行难。加强县（市）城和中心镇规划建设，强化产业支撑，完善服务功能，加快县域城镇化进程。进一步提升管理水平。落实"公交优先"举措，加强市区交通管理，提高公交出行率。创新城市管理体制机制，全面推行市容环卫责任区管理，深入开展城市环境综合整治。进一步加大城市创建力度，力争成为国家卫生城市，力争园林城市通过国家级考核验收；确保获得省级历史文化名城称号，完成国家历史文化名城申报工作。

　　四、加快和谐社会建设

　　（一）着力加强社会事业建设，促进经济社会协调发展。优先发展教育事业。更加注重教育的普惠性，合理配置公共教育资源，促进义务教育均衡发展，力争所有中小学通过省合格学校验收。深入推进素质教育，打造创新教育特色品牌。提高普通高中办学质量，确保三星级以上学校达到70%。大力发展城乡职业教育和培训网络，为产业发展培养更多高素质技能型人才。进一步办好高等教育、成人教育、幼儿教育和特殊教育。继续支持南通大学加快发展。切实加强公共卫生和基本医疗服务。坚持公共医疗卫生的公益性质，深化城乡医疗卫生体制改革，加速公共卫生体系建设，强化重大传染病预防控制。深入实施农民健康工程，提高农村新型合作医疗筹资标准和补偿水平。大力发展城乡社区卫生服务，城市、农村社区卫生服务中心建成率分别达到85%和90%。严格卫生行业监管，深入开展"无红包医院"创建活动，积极构建和谐医患关系。大力发展文化事业和产业。深化文化体制改革，完善公共文化服务体系，完成第二批乡镇文化站建设，扩大公益性文化设施免费开放范围。打造特色文化品牌，丰富群众文化生活。加快培育文化主导产业。搞好文物和非物质文化遗产普查、利用工作。推进有线电视与有线广播共缆传输双入户工程，实施数字电视整体转换。积极开展全民健身活动，发展体育产业。不断提高计划生育优质服务水平。进一步重视发展老年人和残疾人事业，切实保障妇女儿童合法权益。继续加强统计、档案、地方志、保密、气象、无线电管理、关心下一代等工作。

　　（二）着力加强环境保护和建设，启动生态市创建。以生态市创建为抓手，建立健全促进可持续发展的长效机制。实施最严格的环保制度。坚持以集约发展促进污染集中控制，以结构调整促进污染减排。严格建设项目管理，提高环境准入门槛。全面开展化工行业污染专项整治，继续推进重点地区、重点行业污染治理。建立健全环境价格体系，加强对企业行为的外部约束，进一步完善环保执法联动机制，坚决打击各类破坏和污染环境的违法行为。推进生态建设和修复。实施严格的生态功能区划，以水资源和饮用水源保护、土地资源有效供给与保护、森林资源保育与自然保护区建设、湿地与生物多样性保护为重点，推进生态环境保护、建设和修复，构建可持续发展的资源保障体系。加快沿江、沿海、沿河、沿路生态带建设，提高森林覆盖率和绿化覆盖率。积极开展多形式的创建活动。巩固提高创模成果，力争建成全省江北第一个环保模范城市群；以国家级环境优美乡镇和生态村创建为基础，推动各地争创生态县（市）；系统开展绿色社区、绿色家庭、绿色学校和绿色饭店等基层创建活动。

　　（三）着力加强社会管理，保持社会安定有序。按照"党委领导、政府负责、社会协同、公众参与"的总要求，提高社会管理水平。切实加强社区建设。创新社区管理体制，加强社区设施建设和资源整合。将优化行政事务和完善生活服务有机结合，在基本建成社区卫生服务中心的基础上，加快建立一门式综合受理的社区事务服务中心、社区生活求助联动中心，使社区居民办事更方便、困难有人帮。继续加强社区专业社工服务中心建设。大力推进"和谐社区示范单位"创建活动。在外来务工暂住人口相对集中区域建立管理服务中心，实施"三集中"管理服务。积极完善社会组织。加大扶持引导力度，大力培育发展行业协会、商会和社区民间组织等社会组织。抓好社会组织规范化建设，提高自律性和诚信度。加强社会组织分类监管，引导社会自助、自治类组织当好社会"安全阀"。深入创建平安南通。切实加强基层基础工作，拓宽社情民意表达渠道。进一步健全大信访工作格局，认真抓好"三无"示范县（市）区和"四无"示范乡镇创建活动。加大社会治安综合治理力度，创新完善社会矛盾纠纷大调解机制和社会治安大防控体系，依法严厉打击各种违法犯罪活动。层层落实安全生产责任制，依法加大安全生产基础设施投入，着力提高全民安全生产意识和技能，落实安全生产长效机制，坚决防止重特大安全事故发生。继续做好人民防空、国家安全、防震减灾工作。进一步整顿和规范市场秩序，健全立体化价格监管服务体系，切实保障人民群众正当权益。

　　（四）着力培育和谐文化，加强民主法制建设。以社会主义核心价值体系引领社会思潮，营造共建共享和谐的社会环境。进一步提升社会文明。大力弘扬"包容会通、敢为人先"的新时期南通精神和精神文明"南通现象"，全面推进以城市、行业、社区、窗口和村镇为重点的文明创建活动。深入贯彻《公民道德建设实施纲要》，树立、践行社会主义荣辱观，加强未成年人思想道德建设和青年思想政治工作。以培育良好的企业信用和个人信用为重点，加快社会信用体系建设，全力打造"诚信南通"。进一步发扬社会民主。积极支持城乡居（村）民依法自治，保障基层群众依法行使民主权利。全面贯彻《中华人民共和国各级人民代表大会常务委员会监督法》，严格执行人大及其常委会的决议、决定，认真落实审议意见，及时主动报告工作。积极支持政协履行职能，加强与各民主党派、工商联和各人民团体的联系合作。高度重视办好人大议案、政协建议案和人大代表建议、政协委员提案，诚恳听取批评、意见，自觉接受人大、政协监督。进一步做好民族、宗教和侨务、对台工作。进一步健全社会法制。深入实施《法治南通建设实施纲要》，全面推进政治、经济、文化、社会领域的法治化。扎实开展法治江苏合格县（市）区和法治南通合格乡镇（街道）创建活动，力争65%的村（社区）成为民主法治村（社区）。继续抓好"五五"普法，提高全民法治观念和法律素质。进一步强化国防后备力量建设，支持驻通部队建设，继续创建全国双拥模范城。

　　（五）着力加强民生保障，解决好事关人民群众切身利益的现实问题。继续促进城乡统筹就业，不断提高城乡居民收入。积极抓好全国城乡统筹就业试点，构建普惠、长效的促进就业政策体系，实现经济社会发展与扩大就业良性互动。强化就业服务体系建设，实现县（市）乡（镇）劳动力市场信息全部联网，开工建设市人力资源市场。完善就业援助机制，健全职业技能培训制度，重点解决好双失业、零就业家庭的就业困难，做好大专院校毕业生的就业促进工作。完善与经济增长相适应的收入增长机制，规范企业用工行为和工资福利支付，加大执法监察力度，切实维护劳动者特别是农民工的合法权益，构建和谐劳动关系。增加群众投资性、经营性、资产性收入，提高低收入者收入水平，推动共同富裕。积极扩大社会保险覆盖面。加大扩面征缴力度，将被征地农民和进城务工农民逐步纳入城镇社会保险体系，将各项社会保险覆盖到城镇各类企业职工、个体工商户、灵活就业人员。全面建立新型农村社会养老保险制度。切实管好用好社会保险基金，确保安全完整、保值增值。努力维护和促进社会公平。加速构建多层次住房保障体系，继续千方百计解决好城镇中低收入家庭的住房困难。健全城乡居民最低生活保障标准自然增长机制，完善失地农民生活保障制度，提高农村五保户供养水平和集中供养率。加快建立以医疗、教育、司法、灾害救助为主体，其他救助和社会救济、帮扶为补充的城乡社会救助体系。重视社会公益项目建设，鼓励、扶持社会力量兴办福利和慈善事业。进一步做好扶贫和老区开发工作。继续为民办好实事。⑴城镇充分就业社区达55%，帮助2万名城镇失业人员实现再就业；新增农村劳动力转移5.5万人。⑵新型农村合作医疗覆盖率达92%；建立市区城镇非职工居民基本医疗保险制度。⑶农村五保供养标准提高10%，改扩建农村敬老院25所；市区新增老年活动场所10个，新建市老年福利中心。⑷对全市1996年1月以来持独生子女光荣证退休的企业职工实行一次性奖励，其中市区全面完成。⑸完成西北片区域供水工程；完成港闸区自来水老管网改造。⑹改扩建农村桥梁500座，新建和改造农村公路500公里、经济薄弱村硬质机耕路250公里。⑺疏浚整治农村河道238条段，完成土方2293万方；重点整治374个村的河塘，完成土方1798万方。⑻市区建设食品安全动态监控系统，重点对5大类食品7项指标进行定期检测；实施"放心粮油"销售工程，建成质量、卫生、价格、计量、服务"五放心"粮油销售店50家，其中市区20家。⑼市区新建低价位商品房60万平方米；继续安排3000万元为市区住房困难家庭提供政策性住房保障；市区提供套型建筑面积90平方米以下的商品房6000套以上。⑽市区完成30个老小区污水截流工程；实施40个老小区安全防范改造工程。⑾市区新辟、优化公交线路16条，新增、更新公交车辆100辆以上，免费办理公交IC卡。⑿启动市区十字街综合改造工程；市区结合人防工程，建设地下停车场1万平方米，缓解老小区停车难问题。

　　五、加强政府自身建设

　　贯彻落实胡锦涛总书记在中纪委第七次全会上提出的"全面加强新形势下的领导干部作风建设"的要求，全面提高各级政府和领导干部践行科学发展观的能力，推进全面小康、全面腾飞的能力，构建社会主义和谐社会的能力。进一步建设服务政府。牢记根本宗旨，时刻摆正自己和人民群众的位置，继续争创"全省最佳办事环境"，为人民群众多办实事好事，为各类市场主体创造公平竞争环境，全心全意为群众服务、为基层服务、为发展服务。以推进并联审批和网上审批为重点，进一步完善行政审批制度，提高服务效能。创新服务手段，加快建设"数字南通"，启动电子政务二期工程。进一步建设责任政府。坚持科学的发展观和正确的政绩观，把政府追求的目标和人民群众的期盼一致起来，把政府考核的指标和人民群众的评价统一起来，把对上级负责和对人民群众负责结合起来，进一步增强政府的执行力和公信力。健全对涉及经济社会发展全局重大事项决策的协商和协调机制，健全对专业性、技术性较强重大事项决策的专家论证、技术咨询、决策评估制度，健全对与人民群众利益密切相关重大事项决策的公示、听证制度，健全政府新闻发言人制度，扩大公民知情权、参与权、监督权。完善政府责任体系，着力强化资源节约、环境保护、公共安全等方面责任的落实，实行行政问责制、责任追究制。建立和完善重大危险源、应急资源信息管理系统及数据库，认真落实各级各类应急预案，全面提高应急处理能力。进一步建设法治政府。坚持依法行政，加强政府法制建设，依法决策和管理经济社会事务，依法调整各种利益关系，依法解决各种矛盾和问题。完善规范性文件的听证论证、备案审查和依法公示制度。加快建设权责明确、行为规范、监督有效、保障有力的行政执法体制。健全行政执法责任制度，注重行政程序建设，规范自由裁量行为，突出抓好重点领域的执法检查和专项治理。继续做好相对集中行政处罚权工作，推进综合执法试点。坚持抓好行政复议和法制监督工作。进一步建设廉洁政府。牢记"两个务必"，坚持艰苦奋斗，厉行勤俭节约，坚决反对铺张浪费、大手大脚和攀比享受，坚决抵制拜金主义、享乐主义和奢靡之风。健全完善秉公用权、廉洁从政各项制度，从源头上预防和治理腐败。全面落实党风廉政建设责任制，坚持"一岗双责"，加强对政府机关和领导干部的监督。推进政务公开规范化、制度化建设，提高政府工作的透明度，保证行政权力在阳光下运行。强化重点领域、重点部门、重点项目审计。推行政府投资项目"代建制"。坚持纠建并举、综合治理，切实纠正与民争利、损害群众利益的不正之风。继续深入开展治理商业贿赂等专项治理工作。规范公务接待行为。继续办好"政风行风热线"，强化社会监督。对违纪违法案件坚决查处，决不姑息。进一步贯彻实施《公务员法》，加强对公务员的教育、培训和管理，建设一支勤奋学习、忠于职守、清正廉洁、作风优良的公务员队伍。

　　各位代表，全面达小康、建设新南通、实现全面腾飞是时代赋予我们的责任。让我们在省委、省政府和市委的正确领导下，高举邓小平理论和"三个代表"重要思想伟大旗帜，全面落实科学发展观，加快构建社会主义和谐社会，同心同德，不懈进取，全力开创我市又好又快发展新局面，以优异成绩迎接党的十七大胜利召开！
